# Two Remarkable Cases of Phthisis

**Published:** 1909-08-07

**Authors:** E. Brown


### The General Practitioner's Column.

[Contributions to this Column are invited, and if accepted will be paid for.]

### TWO REMARKABLE CASES OF PHTHISIS.

By E. BROWN, M.R.C.S., L.R.C.P. (Lond.).

The first case was a young girl of 18 with early involvement of the right apex. There was slight dulness and crepitation over this area, accompanied by cough, expectoration of sputum containing tubercle bacilli, loss of strength and weight, occasional night-sweats, and evening temperatures of 99.5° to 101° F. In short, it was a typical case of early phthisis in a young subject. She was employed in business, but this was immediately given up, and the question was discussed of a sea voyage, residence at Davos or other mountain climate, or some form of open-air treatment. Eventually open-air treatment at a sanatorium was decided on, and this she underwent for three months. At the end of that period, however, there was a further loss of 7 or 8 lbs. in weight, the cough, expectoration, and night-sweats were no better, and the strength was less. As she was obviously not even maintaining her ground she returned home. Various drugs were tried for some time without lasting benefit. Guaiacol in capsule form seemed to do a little good, but it had to be abandoned after a short trial, as it interfered with digestion and caused sickness. I heard, however, of guaiacose, which contains guaiacol in a non-irritating form, and immediately prescribed it. Very soon the patient began to regain her appetite, and her digestion improved. She took the preparation well from the commencement, and the gradual loss of flesh ceased. In three or four months' time she was putting on weight slightly, while the night-sweats and temperatures had disappeared. She was residing for over a year in a cottage situated on high ground, and though living largely in the open air no special details of that form of treatment were followed. At the end of six months' time she had gained 7 or 8 lbs. in weight, had a good appetite, and all that troubled her was a slight cough. Expectoration was practically nil. When I saw her last she weighed over a stone more than she had ever done before, was able to take long walks and to cycle; in fact, she was enjoying normal health. All that could be detected on examination was a slight occasional "crick" over the right apex on a very deep inspiration. She has since gone to Virginia, U.S.A., and the last report was "perfectly well and strong." The case was under observation for nearly two years from the commencement to the time of writing. In the early phases of her case she must have taken gallons of cod-liver oil and various tonics all to no purpose. If the case is not a "cure" in the strict sense of the term, there does appear to be an absolute arrest of the tubercular process.

The results in the second case were even more striking than in the first, as the latter was just the type in which, under favourable surroundings, one hopes for a cure or, at least, a very marked arrest of the disease. Case II. was one of phthisis in a lady of 63 involving a great part of the left lung, physical signs being present from apex to base, and also the upper lobe of the right lung. Though the disease had obviously been present for some considerable period, she was under no direct treatment, and had not been confined to bed, except for intercurrent attacks of bronchitis, when I first saw her in January 1909. She was then in bed, with great dyspnœa on the least movement and some superadded bronchitis. She had been nursing her daughter for some slight ailment and had broken down under the exertion. There was considerable pain over the left lower ribs in the axillary region, evidencing the presence of pleuritic patches. Shortly after I first saw her she had a hæmorrhage from the lungs, and for a week was dangerously ill. The ordinary treatment of hæmoptysis was followed, and under careful nursing she gradually recovered, but the following symptoms persisted: Dyspnœa, extreme weakness, absolute loss of appetite, cough and expectoration, and dyspepsia on taking any solid food. I put her on guaiacose as soon as the hæmorrhage had subsided, and have persisted in its use to the present time. By Easter she was able to get up a little each day, although she still had no appetite and her condition as to strength remained stationary. Then, with occasional set-backs she began to improve. In a month's time she was able to get out for a little while in a bath-chair, the appetite began to return, the digestion improved, and the cough and expectoration greatly diminished. At the time of writing she is able to walk round to my house and back—about half a mile—has no pain, night-sweats, or temperatures, eats and sleeps well, and only has very slight expectoration. She is still taking guaiacose. Though her weight has not been taken, she has obviously gained considerably. Of course, physical signs remain to tell the tale, but hardly ever are any moist sounds to be detected. She is now able to pursue to a great extent her ordinary social life.

Although the case is too advanced for one to be very hopeful eventually, there has been under the use of guaiacose a marked arrest of the disease. These cases seem to afford reasonable ground for the hope that phthisis may be effectually checked by adopting the dual method of treatment supplied by guaiacose. I am well aware that one swallow does not make a spring, but I have been so impressed by the fact that only to the preparation could the improvement in the foregoing cases be attributed that I think it right to publish my experience. I need hardly say that guaiacose is in no way a secret preparation. It is simply a five-per-cent. solution of guaiacol-calcium-sulphonate in liquid somatose. The guaiacol salt is non-irritating, although potent, while the somatose supplied easily assimilated nutriment. The *motif* of the compound is obvious. The system is braced up, the appetite improved, and gradually the digestion of ordinary diet made possible by the food contained, while the specific action of guaiacol in pulmonary conditions is well established.

